# Maraviroc Inhibits SARS-CoV-2 Through Variant-Dependent Effects on Viral Entry and Mpro Activity Using Single-Round Infectious Particle and Virus-like Particle Models

**DOI:** 10.3390/v18080911

**Published:** 2026-08-19

**Authors:** Uyen Nguyen Phuong Le, Po-Ju Chen, Li-Wei Chu, Jane Cynthia Arifin, Chih-Hao Chen, Yu-Hsuan Chen, Wen-Chi Su, Po-Ren Hsueh, Yueh-Hsin Ping, Cheng-Wen Lin

**Affiliations:** 1 Department of Medical Laboratory Science and Biotechnology, China Medical University, Taichung 404328, Taiwan; phuonguyen6894@gmail.com; 2 Department and Institute of Pharmacology, National Yang Ming Chiao Tung University, Taipei 11221, Taiwan; tim2247749.y@nycu.edu.tw (P.-J.C.); 3 Division of Infectious Diseases, Department of Internal Medicine, China Medical University Hospital, China Medical University, Taichung 404327, Taiwan; 4 Graduate Institute of Biomedical Sciences, China Medical University, Taichung 404328, Taiwan; 5 Department of Laboratory Medicine, China Medical University Hospital, Taichung 404327, Taiwan; 6 School of Medicine, China Medical University, Taichung 404328, Taiwan; 7 Institute of Biophotonics, National Yang Ming Chiao Tung University, Taipei 11221, Taiwan; 8 Office of Research and Development, Asia University, Taichung 413305, Taiwan

**Keywords:** SARS-CoV-2, maraviroc, omicron variant, single-round infectious particle (SRIP), virus-like particles (VLP), spike protein (S), main protease (Mpro)

## Abstract

Maraviroc (MVC), a CCR5 antagonist, has been proposed as a potential antiviral agent against SARS-CoV-2; however, its mechanism of action across viral variants remains unclear. Here, we evaluated the antiviral activity of MVC against SARS-CoV-2 wild-type (WT) and Omicron BA.1 variants using single-round infectious particles (SRIPs), virus-like particles (VLPs), and cell-based assays, with a focus on its impact on viral entry and Mpro function. MVC potently inhibited infection of both WT and BA.1 SRIPs in Vero E6 cells, exhibiting EC_50_ values of 0.0065 μM and 0.016 μM, respectively. Time-of-addition assays revealed that MVC primarily targets the early phase of infection, with the strongest inhibition observed at the viral entry stage, while moderate effects were detected during attachment and post-entry stages. Fluorescence-labeled VLP imaging demonstrated distinct entry pathways, with WT predominantly entering via plasma membrane fusion and BA.1 via endocytosis, independent of cell type. MVC altered WT-VLP trafficking by promoting internalization and lysosomal localization, whereas it had minimal impact on BA.1 internalization. In spike-mediated cell–cell fusion assays, MVC preferentially inhibited WT spike-driven syncytium formation but showed limited effects on BA.1 or BA.4 fusion, while more effectively reducing Omicron spike-mediated binding. At the post-entry stage, MVC inhibited SARS-CoV-2 main protease (Mpro) activity, with BA.1 Mpro (P132H) exhibiting greater sensitivity (IC_50_ = 0.496 µM) than WT (1.869 µM). Collectively, these findings demonstrate that MVC exerts variant-dependent antiviral effects by targeting viral entry, modulating trafficking pathways, and inhibiting Mpro activity. This study highlights MVC as a multi-stage inhibitor with differential efficacy against SARS-CoV-2 variants, providing insights into its potential therapeutic application.

## 1. Introduction

Severe acute respiratory syndrome coronavirus 2 (SARS-CoV-2), a positive-sense single-stranded RNA virus of the Betacoronavirus genus, is the causative agent of coronavirus disease 2019 (COVID-19) and has imposed a substantial global health and economic burden [1]. Its genome encodes structural proteins—spike (S), envelope (E), membrane (M), and nucleocapsid (N)—as well as non-structural proteins, including the main protease (Mpro) and RNA-dependent RNA polymerase (RdRp), which are essential for viral replication [2]. Among these, the spike protein is critical for viral entry by mediating receptor binding and membrane fusion. Viral evolution has primarily involved mutations in the spike protein, particularly within the receptor-binding domain (RBD), while alterations in the N-terminal domain (NTD) also contribute to immune evasion [3]. Successive variants have emerged, with the Alpha and Delta variants driving major outbreaks, and the Omicron variant becoming dominant due to enhanced transmissibility and immune escape [4].

Angiotensin-converting enzyme 2 (ACE2) serves as the primary receptor for SARS-CoV-2 entry [5]. Additional host factors, including transmembrane serine protease 2 (TMPRSS2) and cathepsin L (CTSL), facilitate viral entry through two major pathways: TMPRSS2-dependent plasma membrane fusion and endocytosis-mediated entry [6]. In the absence of TMPRSS2, viral entry proceeds via clathrin-mediated endocytosis and cathepsin-dependent activation in endolysosomes, whereas TMPRSS2 enables spike activation at the cell surface, promoting rapid membrane fusion [7]. SARS-CoV-2 infection induces the formation of multinucleated giant cells, syncytia, which contribute to viral spread and pathogenesis. Spike–ACE2 interactions drive cell–cell fusion, a process mediated by TMPRSS2 [8], and syncytium formation has been shown to facilitate viral replication and transmission [9]. Notably, fusogenic activity varies among variants, with Alpha and Delta exhibiting higher fusion capacity, while Omicron BA.1 maintains sustained fusion activity over time [10,11].

Maraviroc (MVC), a CCR5 antagonist approved for HIV treatment, and leronlimab, against CCR5 receptor, treatment reduced SARS-CoV-2 plasma viremia, suggesting that CCR5 may be a potential antiviral candidate against SARS-CoV-2 [12,13]. Computational analyses indicated that MVC has strong binding to viral and host targets, including Mpro, spike protein, and ACE2 [14,15,16]. Clinical observations further suggested that MVC treatment was associated with improved inflammatory markers [17]. These findings suggested that MVC may exert antiviral effects through multiple mechanisms, including modulation of viral entry and protease activity. However, its efficacy against emerging variants, particularly the Omicron lineage, remains unclear.

In this study, we evaluated the antiviral activity of MVC against the SARS-CoV-2 Omicron BA.1 variant in comparison with the wild-type strain. Using single-round infectious particles (SRIPs) and fluorescence-labeled virus-like particles (VLPs) bearing variant-specific spike proteins, we assessed viral entry and replication in relevant cell models. Confocal and super-resolution microscopy were employed to characterize entry pathways, while cell–cell fusion and Mpro activity assays were performed to elucidate the underlying antiviral mechanisms of maraviroc.

## 2. Materials and Methods

### 2.1. Cell Lines and Culture Conditions

Human embryonic kidney (HEK-293T), Vero E6, and baby hamster kidney (BHK-21) cells were maintained in Dulbecco’s Modified Eagle Medium (DMEM; Gibco™, Waltham, MA, USA) supplemented with 10% fetal bovine serum (FBS; Gibco™, Waltham, MA, USA), 100 U/mL penicillin–streptomycin (PS; HyClone™, Logan, UT, USA), and 1 μg/mL amphotericin B (HyClone™, Logan, UT, USA), and incubated at 37 °C in a humidified atmosphere containing 5% CO_2_. Human lung epithelial Calu-3 cells, used for in vitro viral characterization, were cultured in Minimal Essential Medium (MEM; HyClone™, Logan, UT, USA) supplemented with 10% FBS, 100 U/mL PS, 1 μg/mL amphotericin B, 1 mM sodium pyruvate (Gibco™, Waltham, MA, USA), and 0.1% non-essential amino acids (100×; Corning, Corning, NY, USA) under the same conditions. HEK293T-ACE2 cells, HEK293T cells overexpressing ACE2, were used to evaluate receptor usage [18]. Stable Vero E6 and HEK-293T cell lines expressing S_WH1_-E-M or S_BA.1_-E-M proteins, used for generating wild-type (WT) and BA.1 spike-based single-round infectious particles (SRIPs) and virus-like particles (VLPs), were cultured in the same medium with the addition of G418 (0.8 mg/mL or 1 mg/mL) for selection, as previously described [19,20]. BHK-21 cells expressing enhanced green fluorescent protein (EGFP) and either WT or BA.1 spike protein were used for cell–cell fusion assays [21].

### 2.2. Generation of SARS-CoV-2 Single-Round Infectious Particles (SRIPs)

SARS-CoV-2 SRIPs bearing variant-specific spike (S) proteins from the Wuhan-Hu-1 strain or Omicron BA.1 variant were generated using HEK293T-SBA.1-E-M, HEK293T-SWH1-E-M, Vero E6-SBA.1-E-M, and Vero E6-SWH1-E-M stable cell lines. Cells were transfected with 4 μg of pBAC-SCoV-2rep, a SARS-CoV-2 reporter replicon constructed in the pBeloBAC11 plasmid [20], using jetPRIME reagent (Polyplus, Dandenong, South VIC, Australia). Transfection was performed for 96 h in medium containing 2% FBS. Culture supernatants were collected as WT and BA.1 SRIP stocks. Successful production was confirmed by cytopathic effect, nucleocapsid (N) protein expression via immunofluorescence, and TCID_50_ determination as previously described [20].

### 2.3. In Vitro Infectivity Assay

Vero E6 cells were infected with WT or BA.1 SRIPs at a multiplicity of infection (MOI) of 10, as previously described [20], and subsequently treated with MVC at concentrations of 0, 0.1, 1, and 10 μM. After 96 h of incubation, cells were fixed with 4% paraformaldehyde for 30 min at room temperature, followed by permeabilization with 0.1% Triton X-100 and blocking with 1% bovine serum albumin. Cells were then incubated overnight at 4 °C with an anti-nucleocapsid primary antibody (Genetex, Hsinchu, Taiwan), followed by incubation with the appropriate secondary antibody. Fluorescence images were acquired to determine the proportion of N protein-positive cells, which was quantified by normalization to DAPI-stained nuclei using ImageJ version 1.54g. Relative infectivity was expressed as a percentage, with mock-treated infected cells set to 100%, as previously reported [20,22]. The half-maximal effective concentration (EC_50_) of MVC was determined by fitting dose–response curves [20,22].

### 2.4. Time-of-Addition Assay

Vero E6 or Calu-3 cells were seeded in 6-well plates 24 h before infection. The effects of MVC on different stages of viral infection (attachment, post-attachment, entry, and post-entry) were evaluated using WT or BA.1 SRIPs (MOI = 10). For the attachment assay, cells were incubated with virus and MVC at 4 °C for 2 h, followed by washing and incubation at 37 °C for 18 h. For the entry assay, cells were incubated with virus in the presence of MVC at 37 °C for 2 h before washing and further incubation. In the post-entry assay, cells were infected at 37 °C before MVC treatment. After drug exposure, cells were washed and incubated in fresh medium for 18 h. All conditions were analyzed by immunofluorescence detection of N protein, normalized to DAPI staining, EC_50_ values for each stage were determined using curve-fitting methods as described in our prior studies [20,22].

### 2.5. Generation of Fluorescence-Labeled SARS-CoV-2 VLPs

SARS-CoV-2 wild-type (WT) and BA.1 virus-like particles (VLPs) were generated from culture supernatants of Vero E6 and HEK-293T cells expressing spike (S), envelope (E), and membrane (M) proteins, as previously described [19,20]. Cell debris was removed by centrifugation at 9000× *g* for 30 min, and VLPs were subsequently pelleted by ultracentrifugation at 47,000 rpm for 3.5 h using a Beckman 50.2Ti rotor. Pellets were resuspended in HNE buffer and concentrated using 100 kDa ultrafiltration columns (GE Healthcare, Chicago, IL, USA). Purified VLPs were labeled with Atto647N-NHS ester (Sigma-Aldrich, St. Louis, MI, USA) by incubating 100 μL of VLP solution with 2 nmol dye for 45 min at room temperature [19]. Excess dye was removed using a Sephadex G-25 column, and labeled VLPs were detected by a multimode microplate reader (TECAN 200/200Pro, Männedorf, Switzerland) and stored at −80 °C.

### 2.6. Imaging of VLP Entry

Atto647N-labeled VLPs were used to infect HEK293T-ACE2 and Calu-3 cells. Cells were pre-incubated with VLPs at 4 °C for 30 min and shifted to 37 °C. At indicated time points (0, 30, and 60 min), cells were fixed and stained with WGA488 or LysoBrite Red. Confocal imaging was performed using an Olympus FV1000 confocal microscope (Olympus, Tokyo, Japan). Single-virus tracking (SVT) was conducted using a custom dual-fluorescence microscopy system, as previously described [23]. Super-resolution imaging was performed using Lattice Structured Illumination Microscopy (Lattice SIM^2^) algorithm on a ZEISS Elyra 7 microscope (Carl Zeiss AG, Oberkochen, Germany).

### 2.7. Spike-Mediated Binding and Cell–Cell Fusion Assays

Spike-mediated binding and cell–cell fusion assays were conducted according to previous studies [21,24,25]. Briefly, EGFP-expressing BHK-21 cells presenting WT, BA.1, or BA.4 spike proteins, respectively, were co-cultured with a monolayer of HEK293T-ACE2 cells. For the binding assay, both cells were co-incubated in the presence or absence of MVC (2.5–10 μM) at 4 °C for 1 h and washed with PBS before image acquisition. For the fusion assay, cells were treated with MVC after binding and further incubated at 37 °C for 4 h. EGFP-positive cells were imaged and quantified to assess binding efficiency and syncytium formation. The quantification of binding efficiency and syncytial cell formation was performed as previously described [26].

### 2.8. Trans-Cleavage Assay for Mpro Activity

The CFP-MproCS-YFP reporter assay with SARS-CoV-2 Mpro was performed as described previously [22,27]. HEK293T cells expressing the reporter were transfected with WT or BA.1 (P132H) Mpro plasmids and treated with MVC (0.1–10 μM) for 72 h. Cell lysates were prepared, and fluorescence resonance energy transfer (FRET) signals were measured (436–528 nm). Relative enzymatic activity was calculated and used to determine IC_50_ values as described in our prior studies [22,26].

### 2.9. Statistical Analysis

Data were analyzed using one-way ANOVA with Scheffé’s post hoc test or Student’s *t*-test. Results are presented as mean ± SD, and *p* < 0.05 was considered statistically significant.

## 3. Results

### 3.1. MVC Suppresses Infection by SARS-CoV-2 WT and Omicron BA.1

**Figure 1:**
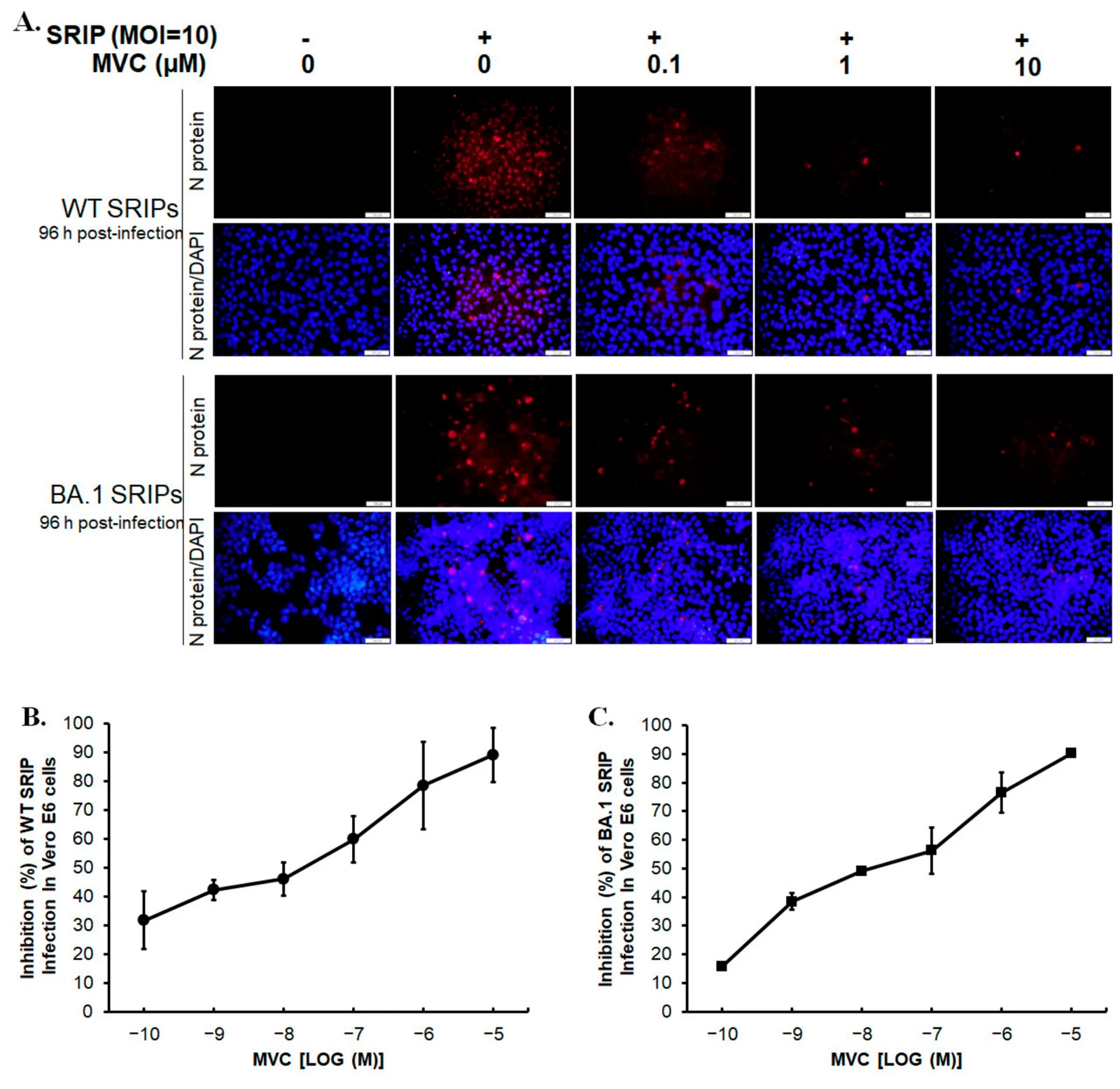
Maraviroc (MVC) inhibits infection by SARS-CoV-2 wild-type (WT) and Omicron BA.1 variants. Vero E6 cells were infected with WT and BA.1 single-round infectious particles (SRIPs) and treated with MVC for 96 h. Infection was evaluated by immunofluorescence staining using anti-SARS-CoV-2 nucleocapsid (N, red fluorescence) antibodies and DAPI (blue fluorescence) (**A**). The percentage of N-positive cells was quantified using ImageJ version 1.54g. Inhibition rates for WT and BA.1 SRIPs. Scale bar: 50 μm. (**B**,**C**) were calculated relative to mock-treated infected cells (set as 100% infectivity) and used to determine the half-maximal effective concentration (EC_50_) values for WT and BA.1 SRIPs.

To evaluate the antiviral activity of MVC against SARS-CoV-2 wild-type (WT) and Omicron variants (BA.1), Vero E6 cells were infected with either WT or BA.1 SRIPs in the presence or absence of MVC at different concentrations. In our previous study [22], we determined the EC_50_ value of nirmatrelvir using the same assay platforms employed in the present work, thereby validating the performance and robustness of these experimental systems with a clinically approved SARS-CoV-2 antiviral agent. Infected cells were assessed by immunostaining of the SARS-CoV-2 nucleocapsid (N) protein, which served as a marker of successful viral infection. Fluorescence imaging showed that MVC reduced the proportion of N protein-positive cells in a dose-dependent manner in both WT- and BA.1-infected cells (Figure 1A). EC50 was used to quantify the effects of MVC treatment. EC_50_ of SARS-CoV-2 WT and BA.1 SRIPs were 0.0065 μM and 0.016 μM, respectively (Table 1, Figure 1B,C). These findings demonstrated that MVC exhibits potent antiviral activity against both the SARS-CoV-2 WT strain and the BA.1 variant.

### 3.2. MVC Primarily Targets Early Stages of SARS-CoV-2 Infection

**Figure 2:**
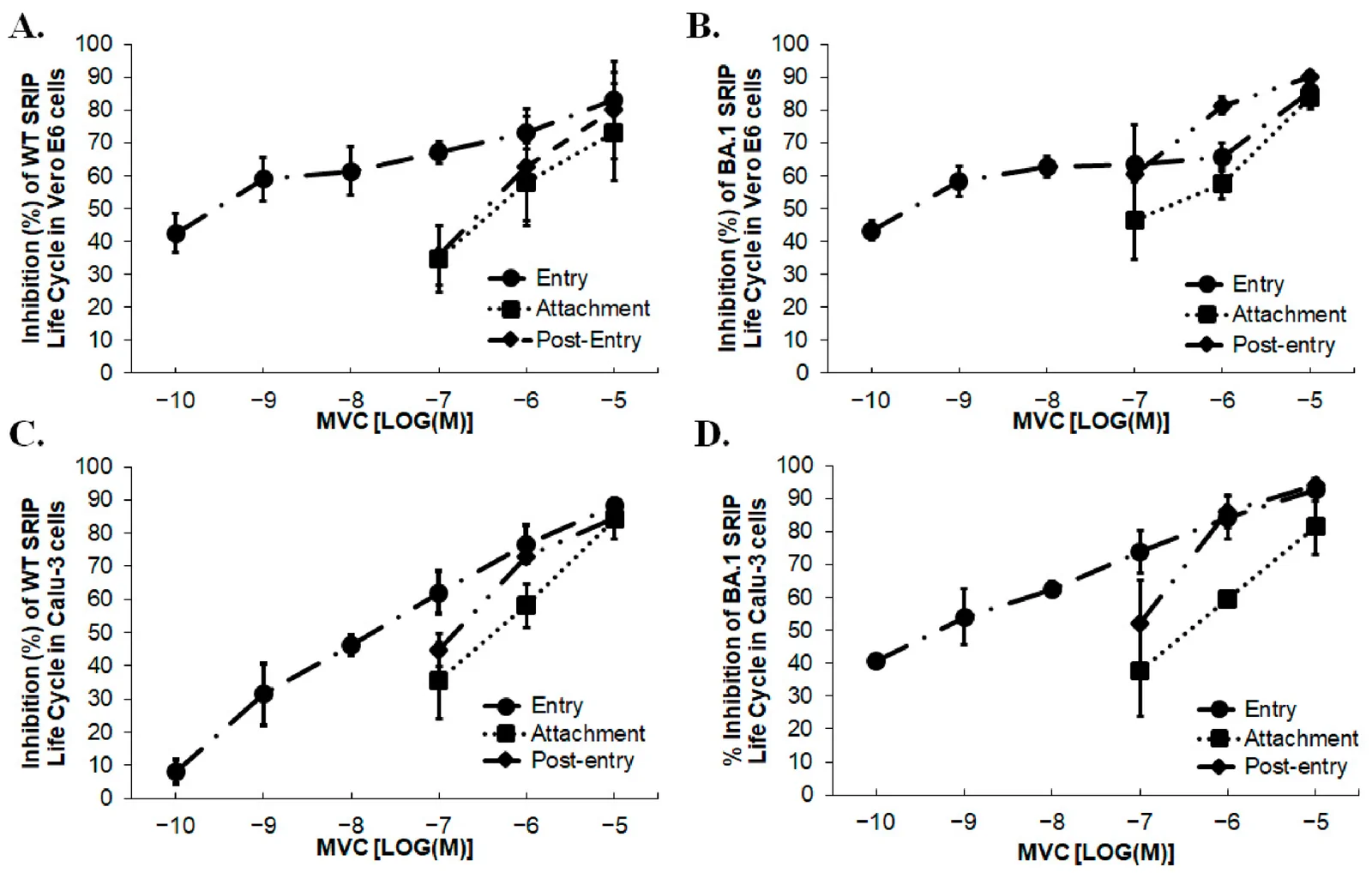
MVC primarily targets the entry and post-entry stages of SARS-CoV-2 infection. Vero E6 (**A**,**B**) and Calu-3 (**C**,**D**) cells were infected with SARS-CoV-2 WT (**A**,**C**) and Omicron BA.1 (**B**,**D**) single-round infectious particles (SRIPs) and treated with various concentrations of MVC at defined time points to assess stage-specific antiviral activity by immunofluorescence assay. For the attachment assay, cells were incubated with virus (MOI = 10) and MVC at 4 °C for 2 h to permit viral binding. For the entry assay, infection was conducted at 37 °C. To evaluate post-entry effects, cells were infected for 2 h before MVC treatment. Nucleocapsid (N) protein expression was quantified using ImageJ version 1.54g, and EC_50_ values were calculated and presented as line graphs (mean ± SD).

The early phase of SARS-CoV-2 infection includes viral attachment, cellular entry, and post-entry processes. To identify the stage at which MVC exerts its strongest antiviral effect, time-of-addition assays were conducted. In Vero E6 cells, MVC showed moderate inhibition during the attachment stage, with BA.1 SRIPs displaying slightly greater sensitivity than WT SRIPs (Figure 2A,B, Table 1). The most potent antiviral activity was observed at the entry stage, particularly for WT SRIPs (EC_50_ = 0.0003 µM), indicating that viral entry is highly susceptible to MVC inhibition. At the post-entry stage, MVC retained moderate activity, with BA.1 SRIPs again showing greater sensitivity than WT SRIPs. In Calu-3 cells expressing detectable levels of CCR5 protein, as confirmed by Western blotting (Figure S2), MVC exhibited comparable inhibitory effects during the attachment stage for both WT and BA.1 SRIPs (Figure 2C,D, Table 1). During the entry stage, MVC remained highly effective, with BA.1 SRIPs demonstrating greater sensitivity (EC_50_ = 0.0006 µM) than WT SRIPs. At the post-entry stage, MVC showed moderate inhibition, with BA.1 SRIPs being slightly more sensitive than WT SRIPs. Overall, these findings indicate that MVC primarily targets the early phase of SARS-CoV-2 infection, particularly the viral entry stage, while exerting moderate inhibitory effects during both the attachment and post-entry stages. Although the magnitude of inhibition varies between WT and BA.1 SRIPs and depends on the cellular context, BA.1 SRIPs tend to be slightly more sensitive to MVC during the attachment and post-entry stages compared to WT SRIPs.

### 3.3. Fluorescence-Labeled SARS-CoV-2 VLPs Reveal Distinct Entry Pathways of WT and BA.1 Variants

**Figure 3:**
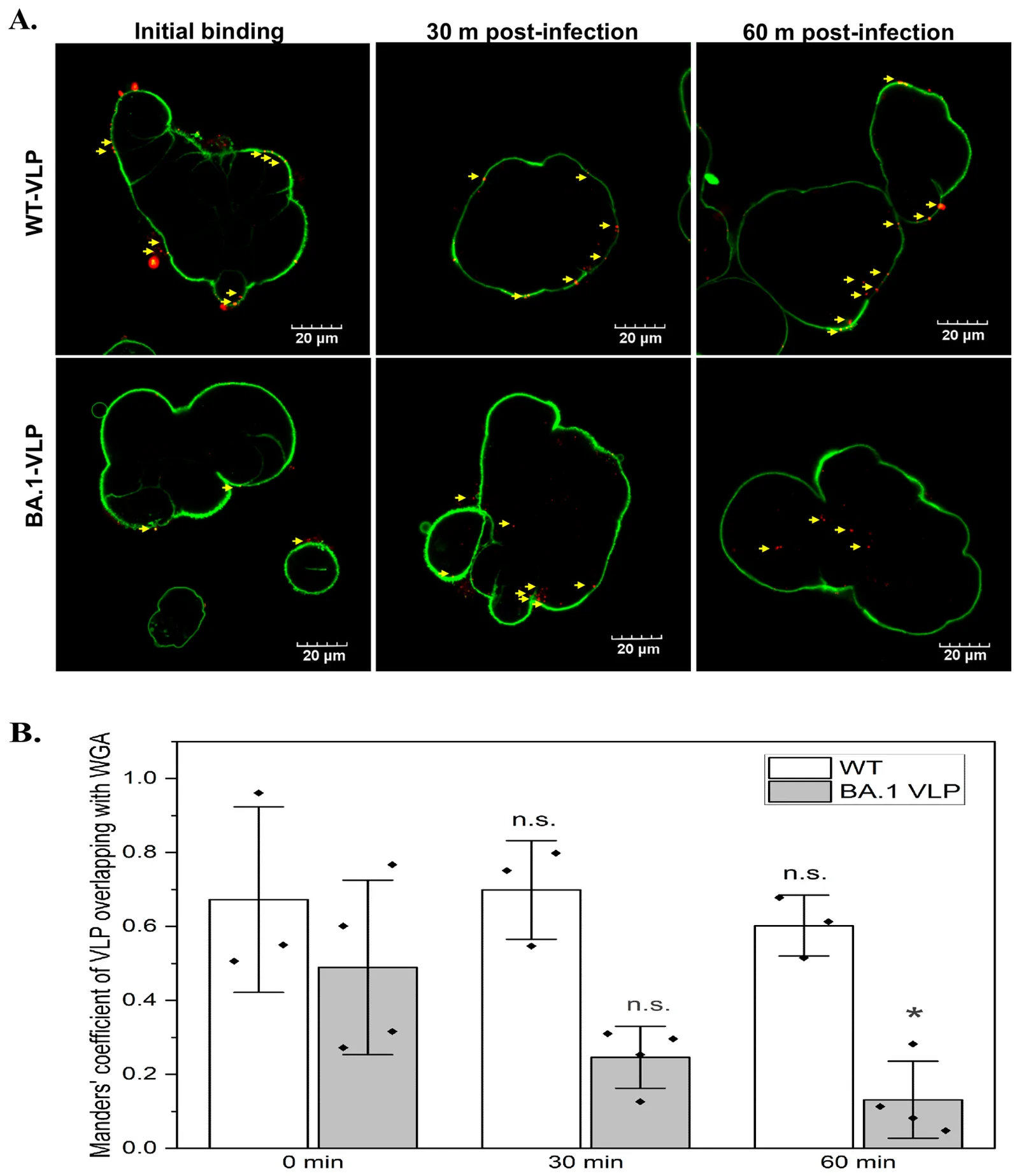

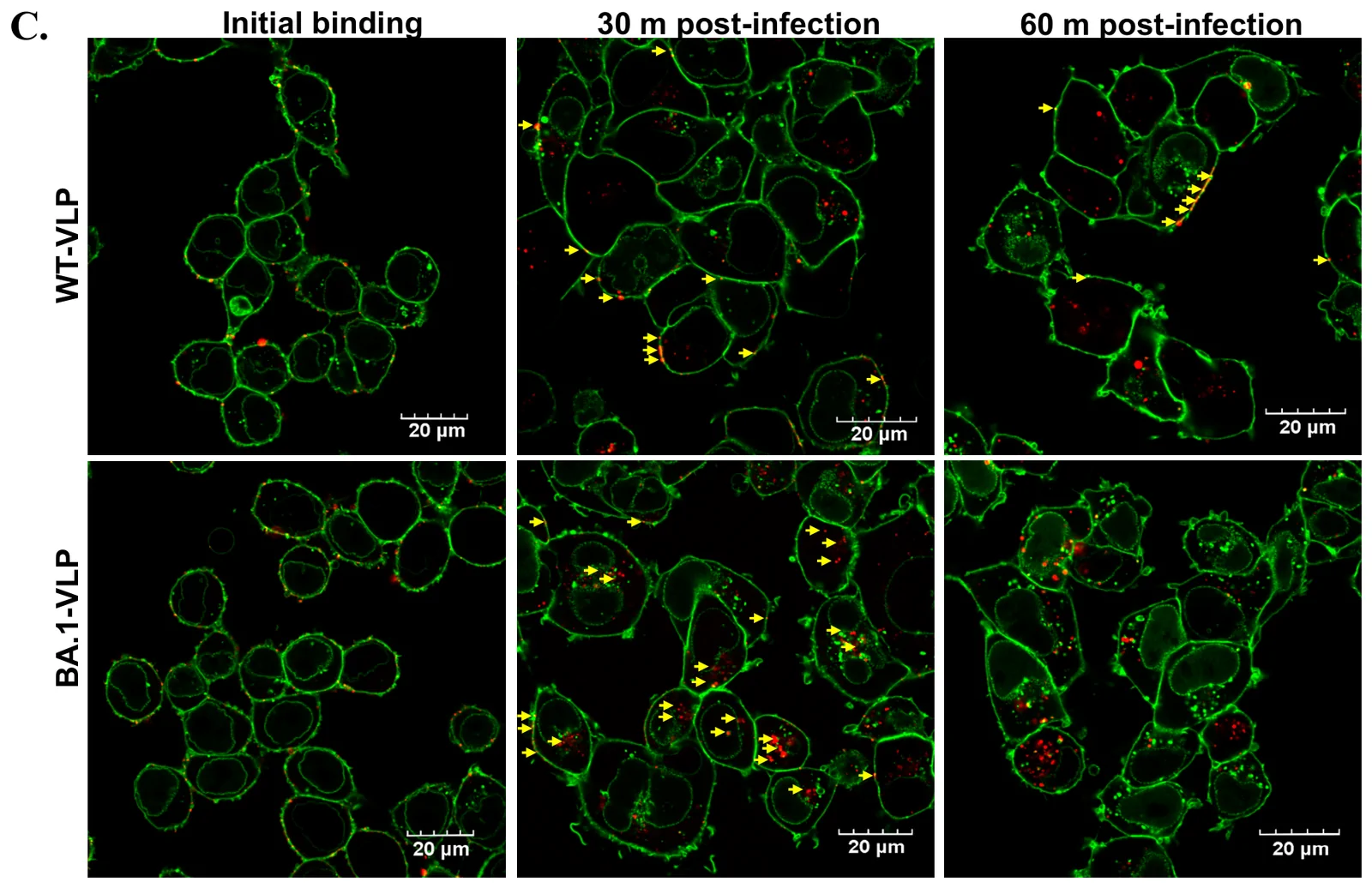
Visualization of WT and Omicron VLP internalization in Calu-3 and HEK-293T-ACE2 cells. Confocal microscopy images illustrate the distribution of WT-VLPs and Omicron VLPs in Calu-3 (**A**) and HEK-293T-ACE2 (**C**) cells at 0-, 30-, and 60-min post infection. Cell membranes were visualized with WGA488 (green), while the VLPs were visualized with Atto647N-NHS (red). (**B**) Quantitative colocalization analysis of the VLPs with the cell membrane in Calu-3 cells. Colocalization of WT-VLPs (white bars) and Omicron BA.1 VLPs (gray bars) with the cell membrane during internalization at 60 min post-infection was quantified. using the Manders’ coefficient (MCC) that represents the fractional overlap of VLP signal with the WGA-labeled membrane area. Scale bar, 20 μm. Yellow arrow, VLPs. *, *p* < 0.05. n.s., not significant.

The difference in MVC sensitivity across SARS-CoV-2 variants (Figure 2) suggested that each variant may rely on a different primary entry pathway. To investigate these viral entry mechanisms, we infected Calu-3 cells with SARS-CoV-2 WT and Omicron BA.1 VLPs. Both VLPs were labeled with the fluorescent dye Atto647N via NHS ester conjugation to E proteins and purified from a G25 column according to our previous study [19] (Supplementary Figure S1), while the Calu-3 cell membranes were stained with WGA488, enabling visualization of VLP internalization. Given that Atto647 was labeled on the envelope of VLPs, if VLPs were entered through membrane fusion, the Atto647 signals of VLPs should be detected on the membrane. Confocal microscopy showed that both WT- and BA.1-VLPs bound on the cell membrane initially (Figure 3A, first column). At 30 min post-infection, Atto647 fluorescence signals from WT-VLPs remained primarily on the cell membrane; only a small fraction of the VLPs was detected inside the cells, even at 60 min post-infection (Figure 3A, top). In contrast, a portion of BA.1-VLPs internalized at 30 min post-infection, and the majority of Atto647 signal was translocated into the cytoplasm by 60 min (Figure 3A, bottom). These results suggest that the WT variant primarily enters via plasma membrane fusion, and the BA.1 variant preferentially utilizes endocytosis. Quantitative colocalization analysis showed that WT-VLPs consistently exhibited more than 50% overlap with the membrane marker WGA488 (Figure 3B, white bars); conversely, BA.1-VLP colocalization decreased over time (Figure 3B, gray bars), reflecting progressive internalization in Calu3 cells.

Given the widespread use of HEK293T-ACE2 cells in studies of SARS-CoV-2 spike-mediated viral entry [19], and the detectable expression of CCR5 protein confirmed by Western blotting (Figure S2), HEK293-ACE2 cells were infected with WT- or BA.1-VLPs to assess whether VLP infection behavior is dependent on cell type. Confocal imaging revealed that Atto647 fluorescence signals of BA.1 were detected mainly in the cytoplasm at 30- and 60-min post-infection, compared to those of WT, consistent with the finding in Calu-3 cells (Figure 3C). Taken together, these results demonstrate that the SARS-CoV-2 VLP infection pathways are variant-dependent and cell type-independent.

### 3.4. MVC Alters WT-VLP Trafficking but Has Limited Effect on BA.1 Internalization

**Figure 4:**
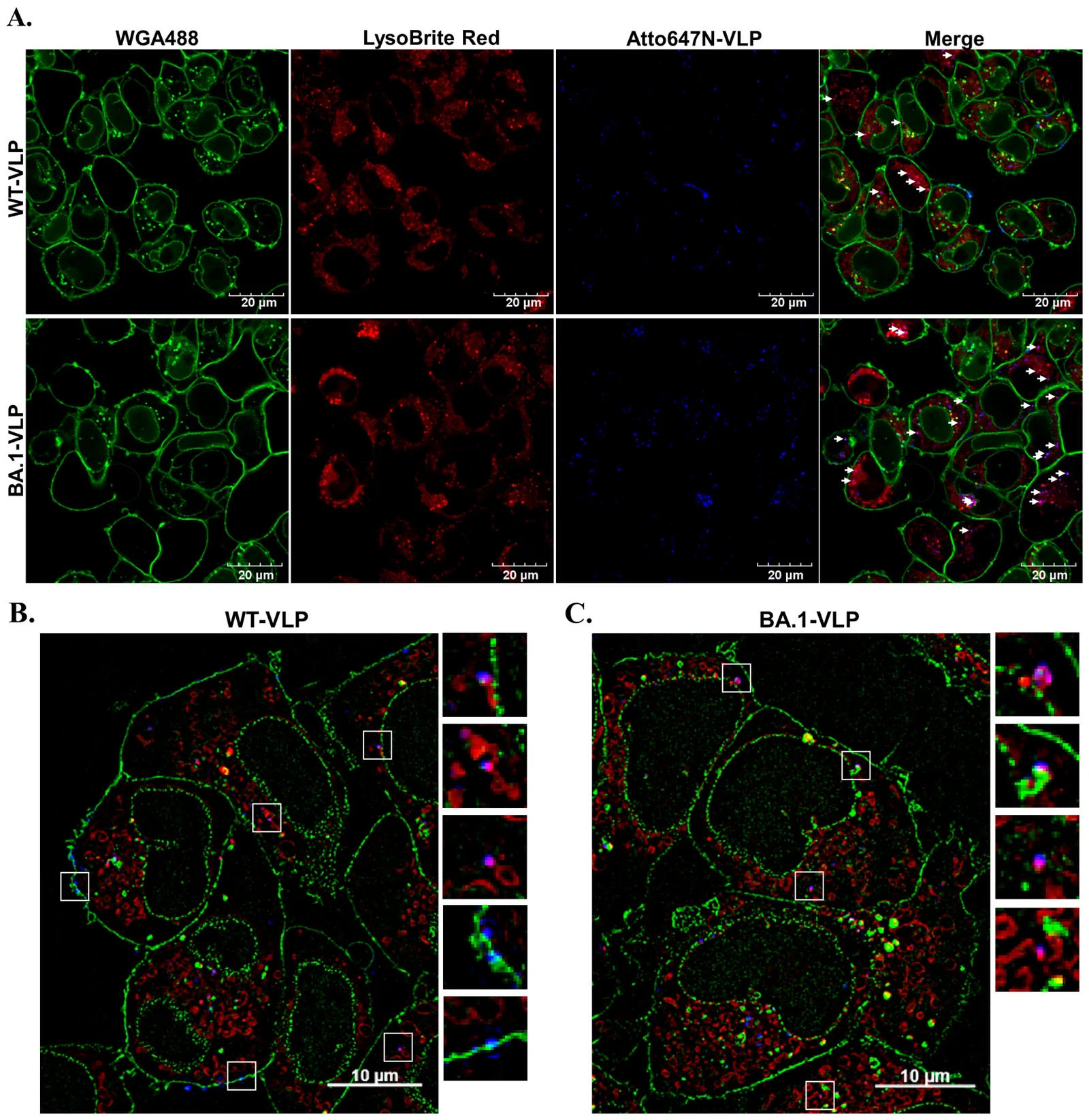
Super-resolution imaging of interactions between WT- or BA.1-VLPs and host cells. Confocal microscopy images showing colocalization of WT- or Omicron (BA.1)-VLPs with cellular compartments (**A**). Cell membranes were stained with WGA-488 (green), acidic organelles (including lysosomes and late endosomes) were labeled with LysoBrite Red (red), and VLPs were labeled with Atto647N. Structured illumination super-resolution microscopy was used to visualize the detailed structure of individual WT- and BA.1-VLPs ((**B**,**C**) respectively). Increased colocalization of Omicron VLPs with acidic organelles was observed in HEK-293T-ACE2 cells. White arrows indicate VLPs localized within acidic organelles. Images were acquired at 60 min post-infection. Red fluorescence: lysosomes. Blue fluorescence: VLPs. Green fluorescence: cell membrane. White square: zoom-in region.

Following internalization, SARS-CoV-2 exploits the acidic environment of lysosomes to facilitate membrane fusion and viral genome release [7]. To investigate the effect of MVC on SARS-CoV-2 trafficking post-internalization, we first assessed whether confocal imaging could reliably visualize the trafficking. HEK293T-ACE2 cells were infected with WT- and BA.1-VLPs, and lysosomes were labeled with LysoBrite-Red. At 60 min post-infection, confocal imaging revealed that both internalized WT- and BA.1-VLPs signals colocalized with LysoBrite-Red, confirming their accumulation in acidic organelles (Figure 4A). Consistent with their higher internalization efficiency (Figure 3), the BA.1 variant showed greater accumulation in these compartments than the WT-VLP variant. Super-resolution SIM imaging further validated the preference of entry pathways for WT- and BA.1- VLPs. The majority of the WT-VLPs appeared to fuse with the plasma membrane; only a subset of WT-VLPs colocalized with acidic lysosomes (Figure 4B, right panel). In contrast, most BA.1-VLPs were detected within lysosomes (Figure 4C, right panel). Notably, the internalized BA.1-VLPs occasionally formed ring-like structures, unlike the internalized WT-VLPs that retained its shape (Figure 4C).

**Figure 5:**
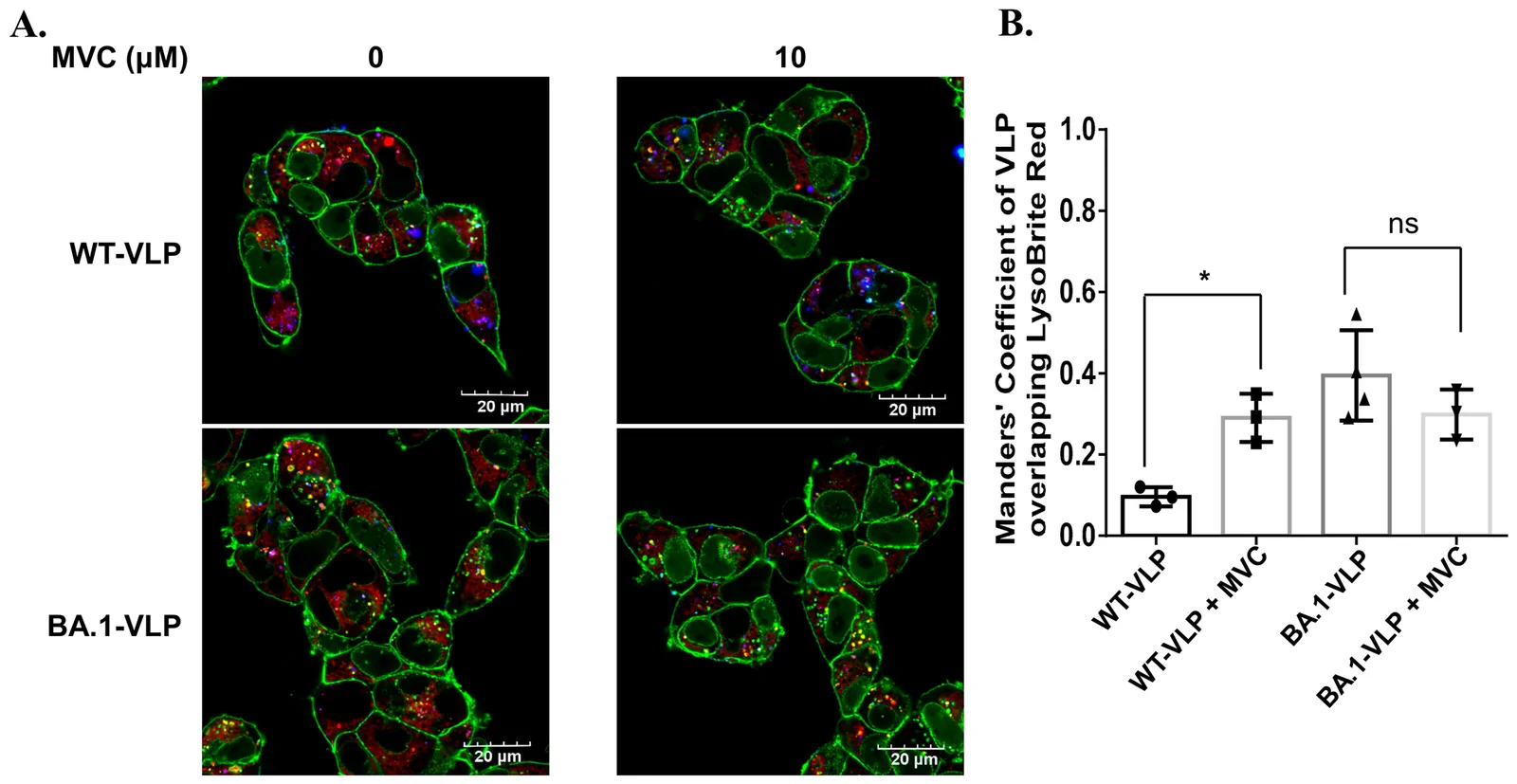
Effect of MVC on interactions between WT- or BA.1-VLPs and host cells. Cells were infected with WT- and BA.1-VLPs and treated with MVC. Cell membranes were stained with WGA-488 (green), and acidic organelles (including lysosomes and late endosomes) were labeled accordingly. Confocal microscopy images show the colocalization of WT- and BA.1-VLPs with acidic organelles at 60 min post-infection (**A**). Differential association of WT-VLPs and Omicron-VLPs with lysosomes and late endosomes in HEK-293T-ACE2 cells was quantified using Manders’ coefficient (**B**). Red fluorescence: lysosomes. Blue fluorescence: VLPs. Green fluorescence: cell membrane. *, *p* < 0.05. ns, not significant.

We then treated HEK293T-ACE2 cells with 10 μM MVC, followed by infection with WT- and BA.1-VLPs (Figure 5A). Confocal imaging revealed that the MVC treatment increased the WT-VLPs internalization and enhanced their trafficking to acidic organelles compared to the control group (Figure 5A, top). Conversely, MVC treatment did not enhance BA.1-VLP internalization and slightly reduced their intracellular abundance (Figure 5A, bottom). Quantitative colocalization analysis confirmed these observations. MVC treatment significantly increased the overlap between Atto647-labeled WT-VLPs and LysoBrite-Red signal; the BA.1-VLP group showed no significant change in colocalization level upon MVC treatment (Figure 5B). Taken together, these findings suggest that MVC alters the entry process of SARS-CoV-2 WT VLPs into the endocytic pathway, but not Omicron BA.1 VLPs.

### 3.5. MVC Differentially Affects WT- and BA.1-Spike-Mediated Binding Attachment and Cell–Cell Fusion

**Figure 6:**
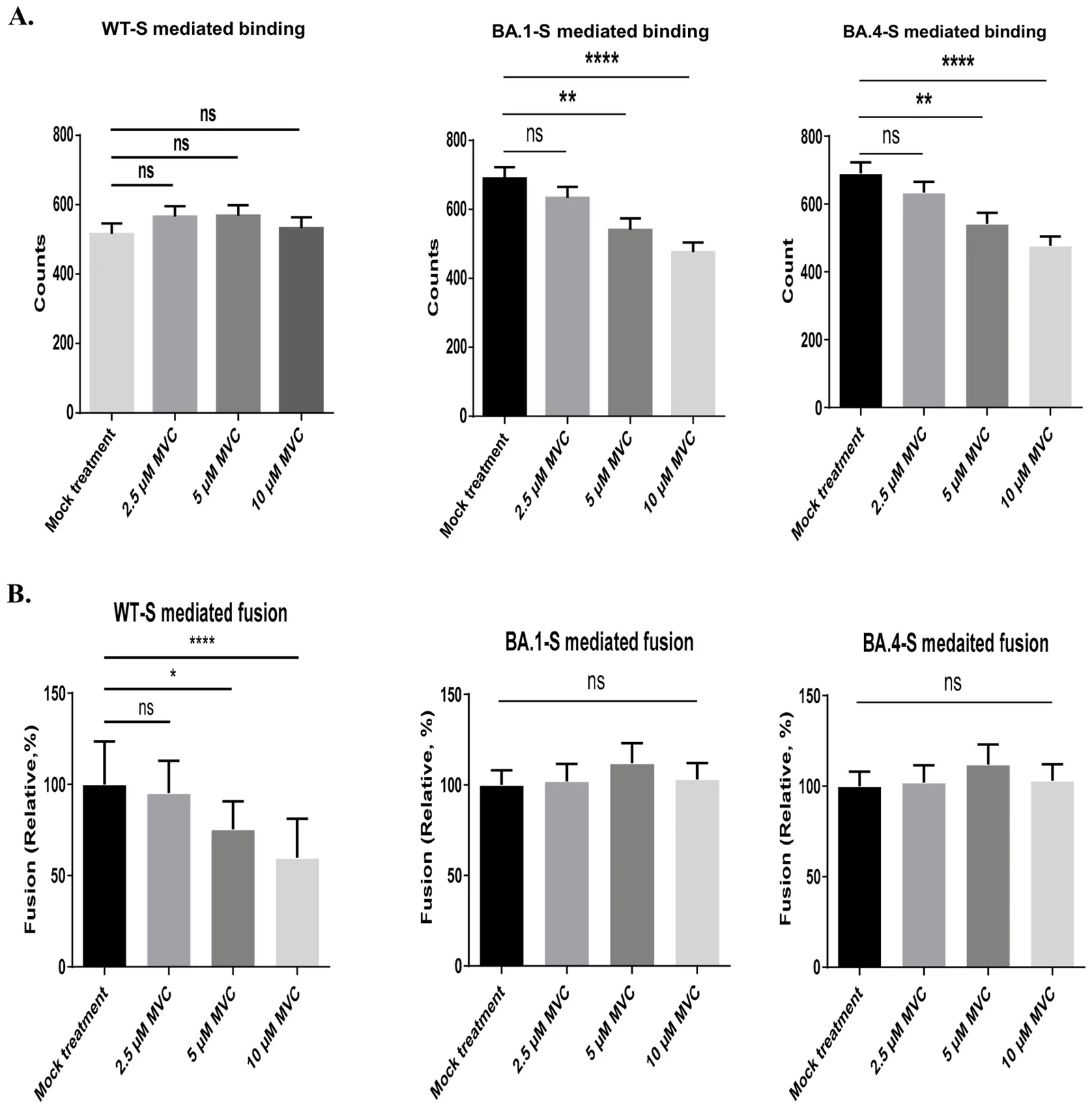
Effect of MVC on WT and Omicron BA.1/BA.4 spike-mediated cell–cell fusion. EGFP-expressing BHK-21 cells bearing WT, BA.1, or BA.4 spikes were co-cultured with HEK293T-ACE2 cells. Binding was assessed after incubation with or without MVC (2.5–10 μM) at 4 °C for 1 h (**A**). Fusion was evaluated following MVC addition and incubation at 37 °C for 4 h (**B**). EGFP-positive cells were imaged and quantified. *, *p* < 0.05. **, *p* < 0.01. ****, *p* < 0.001. ns, not significant.

Given the distinct entry pathways utilized by WT and BA.1 variant, the effects of MVC on spike-mediated binding attachment and cell–cell fusion were investigated using a cell–cell fusion assay [21,25]. A monolayer of HEK293T-ACE2 cells was co-cultured with EGFP-expressing BHK-21 cells bearing WT, BA.1, or BA.4 spike proteins. MVC exhibited minimal effects on WT spike-mediated cell binding but more effectively reduced BA.1- and BA.4-mediated cell binding (Figure 6A). In contrast, MVC markedly inhibited WT spike-mediated syncytium formation in a dose-dependent manner, while showing no significant inhibitory effect on BA.1- or BA.4-mediated fusion (Figure 6B). These findings demonstrate that MVC differentially modulates spike-mediated functions among viral variants. MVC preferentially disrupts WT spike-driven membrane fusion, resulting in a more pronounced inhibition of WT syncytium formation under the experimental conditions. Meanwhile, MVC more effectively inhibits the binding of Omicron BA.1 and BA.4 spikes to ACE2 on HEK293T cells. Together, these results suggest that MVC interaction with CCR5 exerts dual effects, suppressing WT spike-mediated membrane fusion while modulating Omicron spike-mediated ACE2 binding.

### 3.6. MVC Effectively Inhibits Mpro Activity in WT and BA.1 Variants

**Figure 7:**
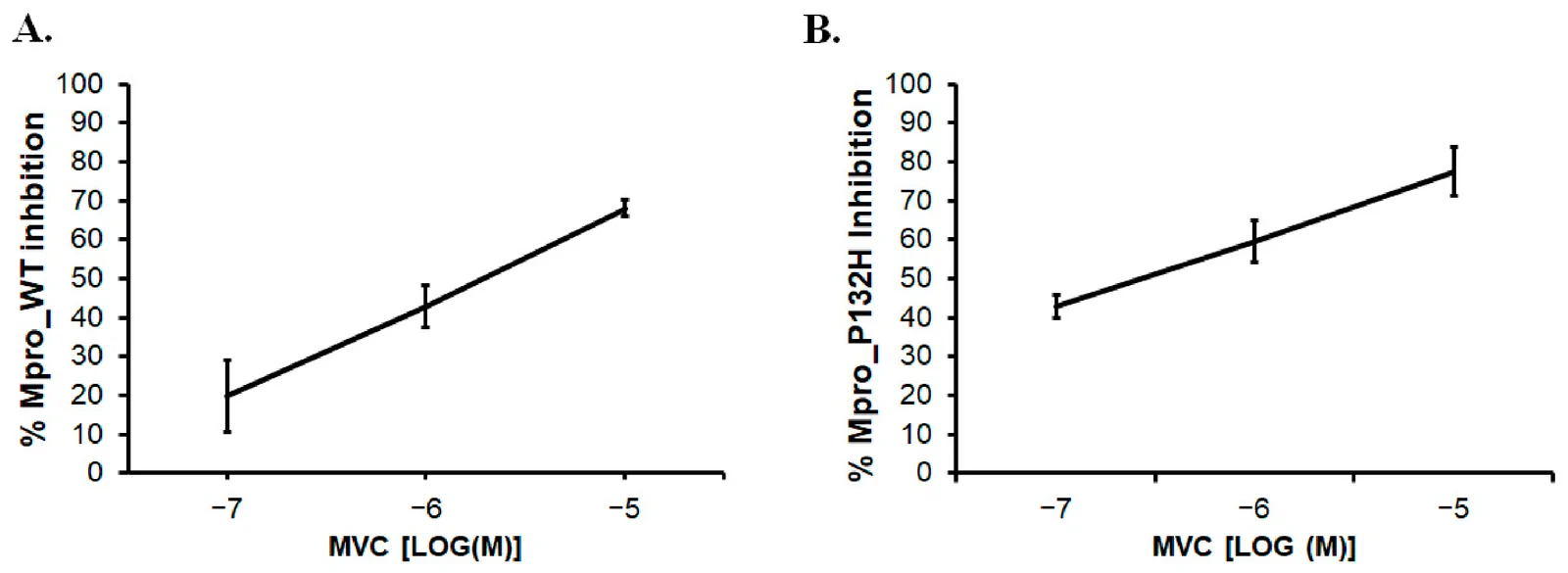
Effect of MVC on trans-cleavage activity of WT and Omicron BA.1 Mpro. The CFP–MproCS–YFP FRET reporter assay was performed as described previously. HEK293T cells expressing the reporter were transfected with Mpro_WT (**A**) or Mpro_BA.1 (P132H) (**B**) and treated with MVC (0.1–10 μM) for 72 h. Cell lysates were analyzed by FRET (436–528 nm), and enzymatic activity was calculated relative to the mock-treated control (100% activity).

To investigate the role of MVC at the post-entry stage of viral replication, plasmids encoding Mpro_WT and Mpro_BA.1 (harboring the P132H mutation) were transiently transfected into the HEK293T_CFP_MproCS_YFP stable cell line in the presence or absence of MVC (Figure 7). Mpro activity was subsequently evaluated using a trans-cleavage FRET assay. The results demonstrated that MVC inhibited Mpro_BA.1 enzymatic activity in a concentration-dependent manner, with an IC_50_ value of 0.496 µM. In comparison, Mpro_WT exhibited a higher IC_50_ value (1.869 µM), indicating that Mpro_BA.1 is more sensitive to MVC inhibition. These findings suggest that the P132H mutation enhances the susceptibility of Mpro to MVC. Consistent with the results shown in Figure 2, these data support the conclusion that MVC exerts inhibitory effects at the post-entry stage of SARS-CoV-2 infection, specifically through targeting Mpro activity.

## 4. Discussion

Maraviroc (MVC), a CCR5 antagonist originally developed to inhibit HIV-1 entry, has recently attracted attention as a potential repurposed antiviral agent for SARS-CoV-2 [17,27]. The major finding of the current study is that MVC displays variant-dependent and stage-specific anti-SARS-CoV-2 activity. In the current study, MVC reduced SARS-CoV-2 N protein-positive cells in a dose-dependent manner in both WT and BA.1 SRIPs, indicating antiviral activity against these variants (Figure 1). Mechanistic analysis showed that MVC exerted its strongest inhibitory effect during viral entry, with moderate effects at attachment and post-entry stages (Figure 2, Table 1). Using fluorescence-labeled VLP imaging, spike-mediated binding and fusion assays, our results depicted that MVC strongly reduced WT spike-mediated cell–cell fusion and altered WT-VLP trafficking toward acidic organelle-associated compartments, whereas Omicron BA.1 and BA.4 spikes were affected in spike-mediated binding and differential post-entry reporter responses (Figure 4 and Figure 5). These findings suggest the antiviral activity of MVC against SARS-CoV-2 infection.

In the present study, VLP-based imaging revealed variant-specific differences in the early entry-associated trafficking of SARS-CoV-2. WT-VLPs predominantly exhibited sustained membrane-associated signals, whereas Omicron BA.1-VLPs showed progressive accumulation within intracellular acidic organelles over time, suggesting that BA.1 relies more strongly on endocytic trafficking than the WT strain (Figure 3 and Figure 4). Notably, treatment with the CCR5 antagonist MVC enhanced the localization of WT-VLPs to acidic organelle-positive compartments, while exerting only a limited effect on BA.1-VLP distribution (Figure 5). These observations suggest that MVC may alter the entry route of WT SARS-CoV-2, shifting viral trafficking from plasma membrane-associated fusion toward an endocytosis-dependent pathway. In contrast, because BA.1 already preferentially utilizes endosomal entry, its intracellular trafficking pattern appears less responsive to MVC treatment. This interpretation is consistent with previous reports demonstrating that Omicron variants exhibit reduced TMPRSS2 usage, decreased spike cleavage efficiency, attenuated fusogenicity, and increased dependence on endosomal entry compared with earlier SARS-CoV-2 strains [28,29]. Furthermore, MVC differentially affected spike-mediated membrane fusion among variants. While MVC inhibited WT spike-driven cell–cell fusion, it showed minimal effects on BA.1- or BA.4-mediated fusion activity (Figure 6), consistent with previous evidence that MVC suppresses WT spike-mediated syncytium formation [30]. Collectively, these findings support a variant-dependent role of CCR5 in regulating SARS-CoV-2 entry and suggest that MVC-mediated inhibition is more pronounced for variants that retain higher membrane fusion activity.

CCR5 antagonists such as MVC and TAK-779 are potent inhibitors of HIV entry, acting by blocking gp120–CCR5 interactions and subsequent viral fusion [31]. MVC binds to a hydrophobic pocket within the transmembrane region of CCR5, locking the receptor in a conformation that fails to recognize distal regions of the Env V3 loop and prevents the conformational changes in gp120 and gp41 required for insertion of the gp41 fusion peptide into the host cell membrane, ultimately reducing viral binding efficiency [32]. These results raise the possibility that stabilization of CCR5 in this fixed conformation by MVC may also be associated with the observed reduction in Omicron spike binding to ACE2, which warrants further investigation.

MVC also inhibited post-entry stages of SARS-CoV-2 replication, suppressing the enzymatic activity of both Mpro_WT (IC_50_ = 1.869 µM) and Mpro_BA.1 (IC_50_ = 0.496 µM) in a dose-dependent manner (Figure 2 and Figure 7). These findings indicate that MVC exerts antiviral effects at the post-entry stage by targeting Mpro activity. Previous virtual screening studies of FDA-approved drugs identified MVC as a potential Mpro inhibitor, capable of binding to the substrate-binding pocket and forming multiple non-covalent interactions with conserved residues [15].

Together, these findings suggest that MVC may serve as a repurposed therapeutic candidate for COVID-19. Its variant-dependent antiviral effects further highlight the potential value of MVC in host-directed strategies tailored to distinct SARS-CoV-2 infectious pathways.

## 5. Conclusions

This study demonstrates that MVC exerts antiviral activity against SARS-CoV-2 through dual mechanisms targeting both viral entry and post-entry stages. Mechanistic analyses show that MVC differentially modulates variant-specific entry pathways, redirecting WT virus toward endocytic trafficking while exerting minimal effects on BA.1 internalization, consistent with the distinct entry features of Omicron variants. In addition, MVC inhibits WT spike-mediated membrane fusion but has a limited impact on Omicron spike-driven fusion, underscoring variant-dependent differences in fusogenicity and CCR5 involvement. Notably, MVC also suppresses post-entry replication by targeting the viral main protease (Mpro), in agreement with prior in silico studies indicating stable binding within the Mpro substrate-binding pocket. Collectively, these findings provide experimental evidence that MVC functions through both host- and virus-targeted mechanisms, supporting its potential as a repurposed therapeutic for COVID-19.

## Figures and Tables

**Table 1:** Half-maximal effective concentration (EC_50_) of MVC on viral infectivity and early replication stages of SARS-CoV-2 wild-type and Omicron BA.1 SRIPs.

	EC_50_ (µM) of MVC	
	WT SRIPs ^a^	BA.1 SRIPs ^a^
In vitro infectivity	0.0065 ± 0.0029	0.016 ± 0.0052
Vero E6 cells		
Attachment	0.63 ± 0.23	0.36 ± 0.05
Entry	0.0003 ± 0.0001	0.0004 ± 0.0002
Post-entry	0.31 ± 0.07	<0.1
Calu-3 cells		
Attachment	0.48 ± 0.38	0.42 ± 0.29
Entry	0.025 ± 0.012	0.0006 ± 0.0003
Post-entry	0.16 ± 0.003	0.109 ± 0.017

^a^ Wu-han-Hu-1 (WT) strain and Omicron BA.1 variant (BA.1) SARS-CoV-2 single-round infectious particles (SRIPs).

## Data Availability

The original contributions presented in this study are included in the article/Supplementary Materials. Further inquiries can be directed to the corresponding authors.

## Supplementary Materials

The following supporting information can be downloaded at: https://www.mdpi.com/article/10.3390/v18080911/s1, Figure S1: Size-exclusion chromatography profiles of SARS-CoV-2 VLPs. Figure S2. Western blot analysis of CCR5 expression in HEK-293T, HEK293T-ACE2, and Calu-3 cells.
