# Supplementary material for: Maraviroc Inhibits SARS-CoV-2 Through Variant-Dependent Effects on Viral Entry and Mpro Activity Using Single-Round Infectious Particle and Virus-like Particle Models
Source: Viruses. 2026 Aug 19;18(8):911. doi: 10.3390/v18080911 (PMC13517540; doi:10.3390/v18080911)
Supplement: Supplementary file 1 [file viruses-18-00911-s001.zip › viruses-4342787-supplementary.pdf]

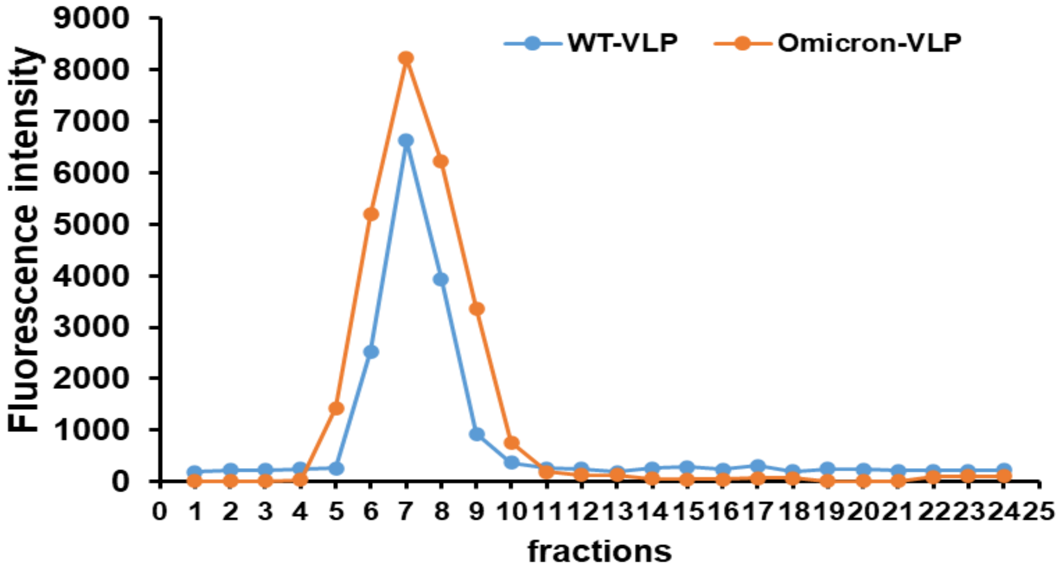

Figure S1. Size-exclusion chromatography profiles of SARS-CoV-2 VLPs. Fluorescence intensity profiles from a Sephadex G-25 column are shown. Atto647N-labeled WT and Omicron BA.1 VLPs are indicated by blue and brown lines (circles), respectively.

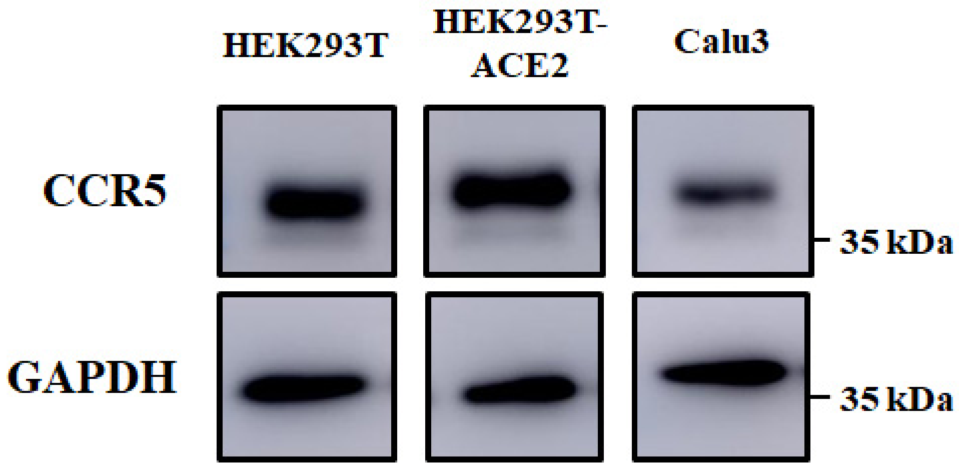

Figure S2. Western blot analysis of CCR5 expression in HEK-293T, HEK293T-ACE2, and Calu-3 cells. Cell lysates were sonicated, clarified, and proteins were separated by SDS-PAGE and transferred to PVDF membranes. After blocking with 5% milk in TBST, membranes were incubated overnight at 4 °C with anti-CCR5 and β-actin antibodies, followed by HRP-conjugated secondary antibodies for 1 h. Bands were detected using LumiFlash™ Prime and chemiluminescence imaging.
